# Supplementary material for: Reduction of Ischemic Stroke Associated Disability in the Population: A State-Wide Stroke Registry Analysis over a Decade
Source: J Clin Med. 2022 Nov 25;11(23):6942. doi: 10.3390/jcm11236942 (PMC9737852; doi:10.3390/jcm11236942)
Supplement: Supplementary file 1 [file jcm-11-06942-s001.zip › jcm-1941697-supplementary.pdf]

Supplementary Materials

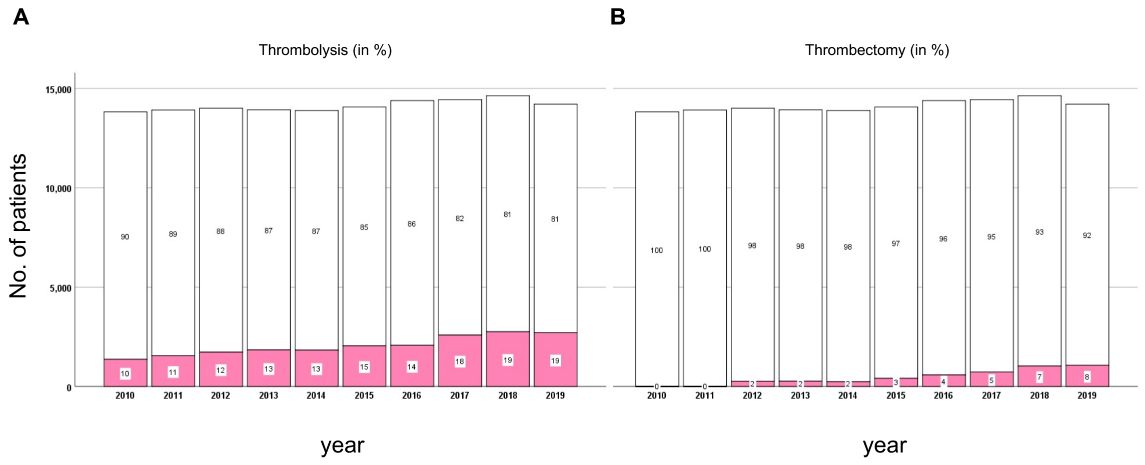

Supplementary Figure S1. Increasing proportions of stroke patients were treated with thrombolysis (**A**) and thrombectomy (**B**) from 2010 to 2019, respectively. The Y-axis shows the total number of admitted patients per year.

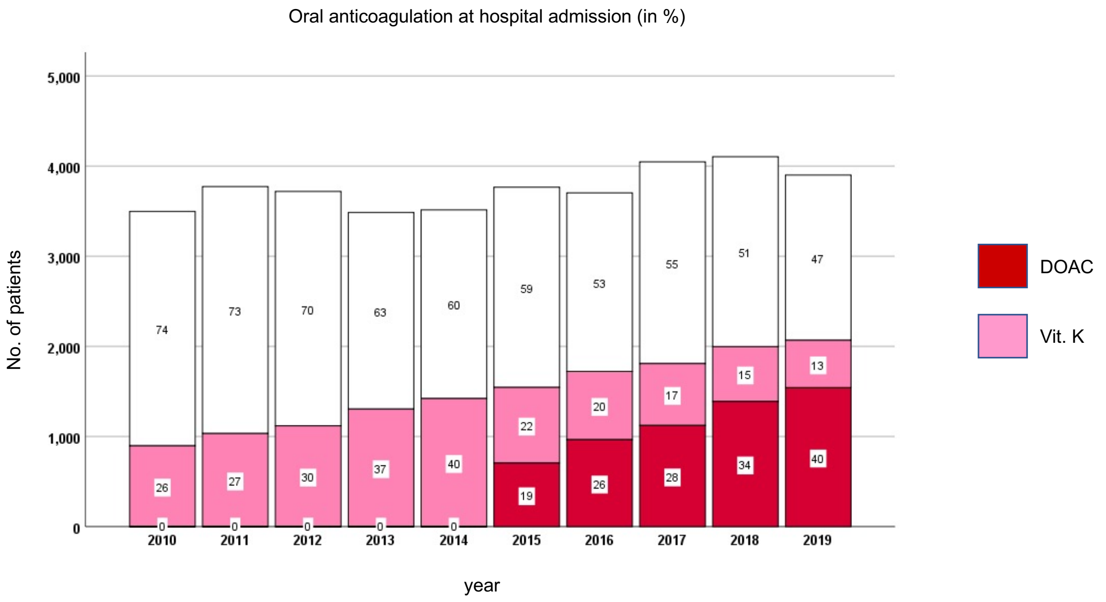

Supplementary Figure S2. Increasing proportion of patients were treated with anticoagulants prior to hospital admission from 2010 to 2019. The Y-axis shows the total number of admitted patients per year.

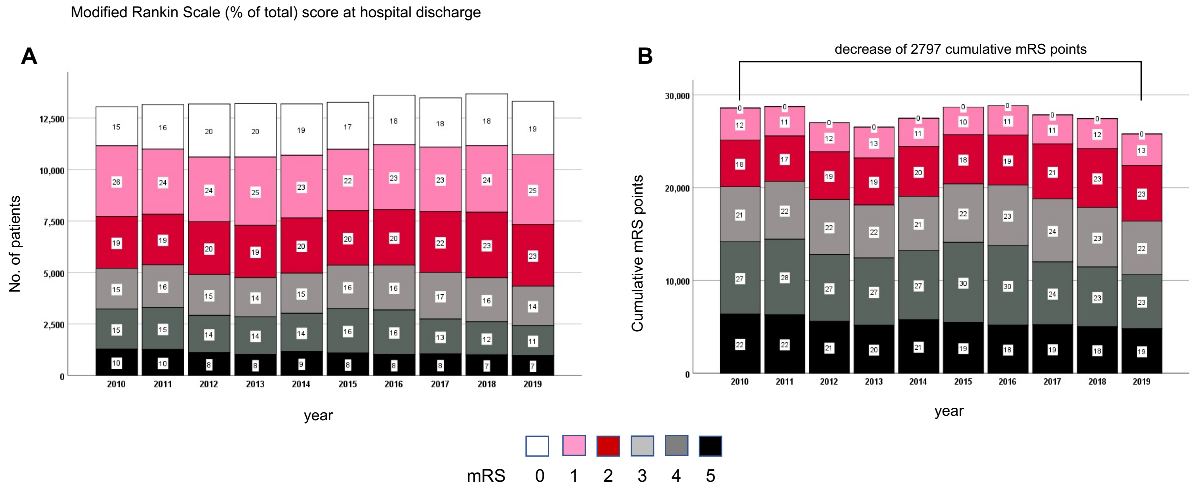

Supplementary Figure S3. (**A**) Whereas the total number of patients remained stable during the study period, the proportion of patients with severe disability at hospital discharge decreased over time. Numbers indicate % of patients in the respective mRS category. (**B**) The cumulative number of mRS points at discharge (as a measure of the burden of stroke disability to bear for the caregiving system) decreased over time. This was due to a reduction of severely disabled cases. 2797 cumulative mRS points were saved in the federal state of Hesse in 2019 in comparison to 2010. Numbers indicate % of cumulative mRS points related to the respective mRS category.

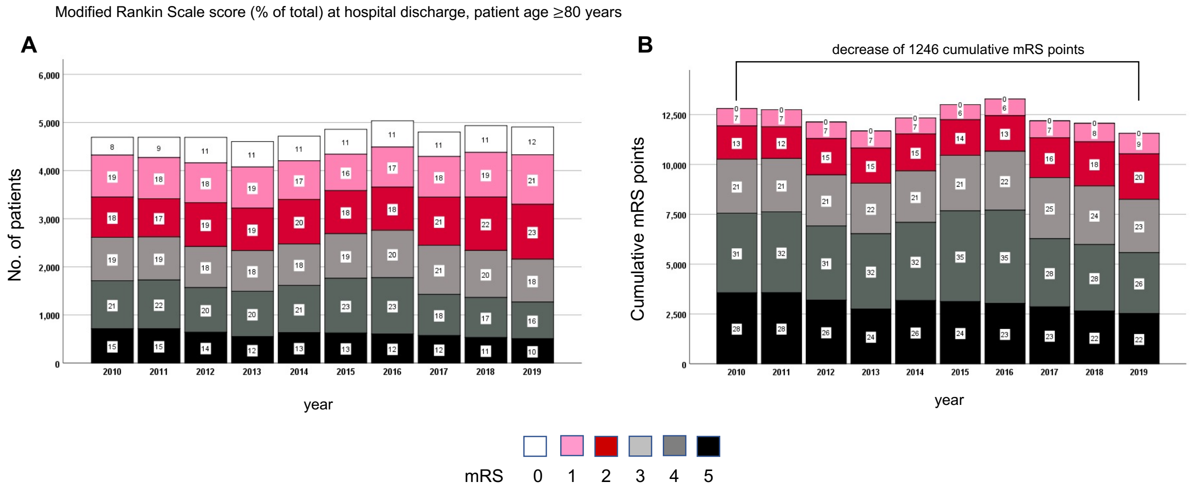

Supplementary Figure S4. The figure only shows data on patients aged 80 years or older at hospital admission. (**A**) The proportion of patients aged 80 years or older with severe disability at hospital discharge decreased over time. Noteworthy, the total number of patients aged 80 years or older remained stable during the study period. Numbers indicate % of patients in the respective mRS category. (**B**) The cumulative number of mRS points at discharge (as a measure of the burden of stroke disability to bear for the caregiving system) decreased over time. Numbers indicate % of cumulative mRS points related to the respective mRS category.

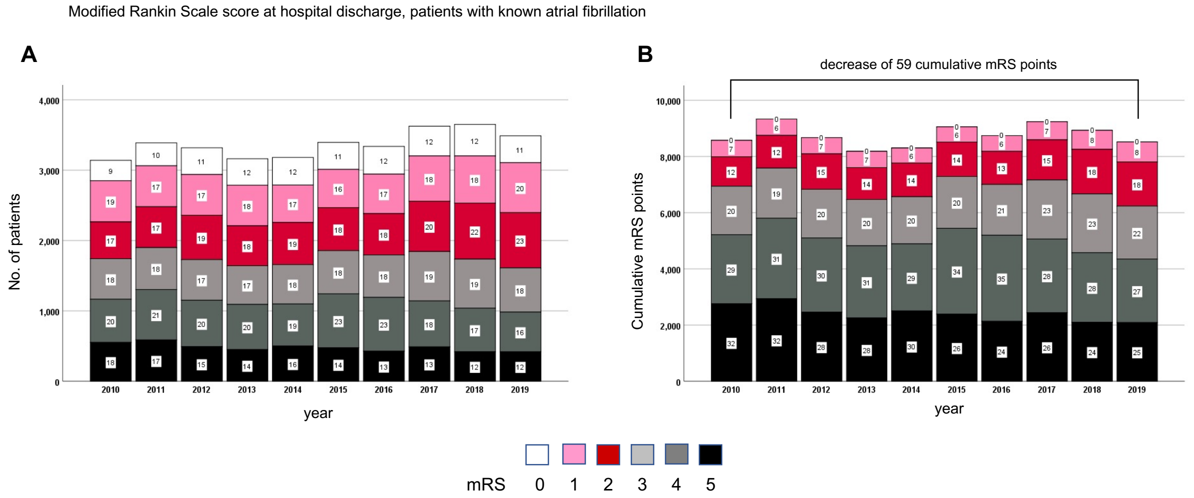

Supplementary Figure S 5. The figure only shows data on patients known atrial fibrillation at hospital admission. (**A**) The proportion of patients with known atrial fibrillation at hospital admission and severe disability at hospital discharge decreased over time. Numbers indicate % of patients in the respective mRS category. (**B**) The cumulative number of mRS points at discharge (as a measure of the burden of stroke disability to bear for the caregiving system) decreased over time. Numbers indicate % of cumulative mRS points related to the respective mRS category.

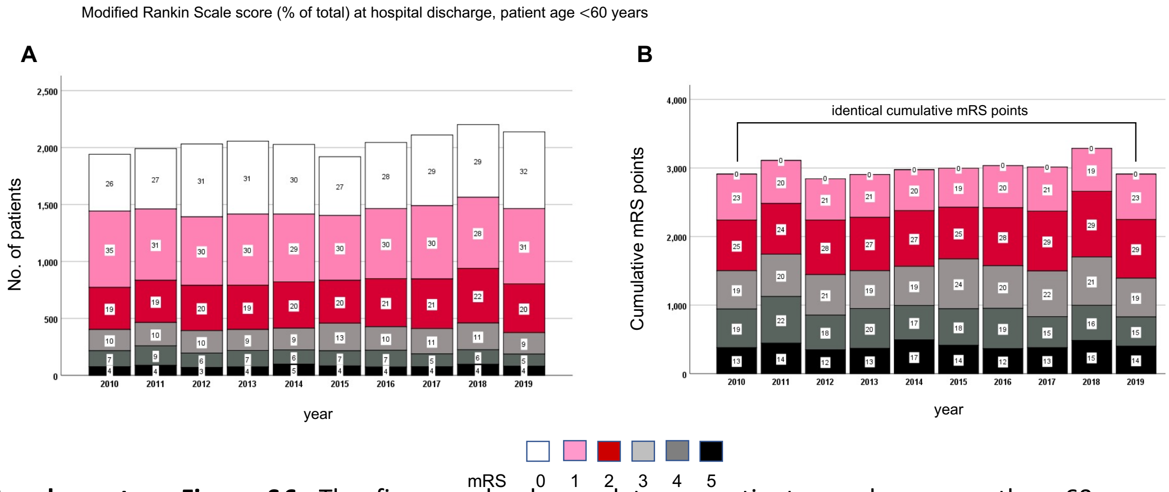

Supplementary Figure S6. The figure only shows data on patients aged younger than 60 years at hospital admission. (**A**) The proportion of patients aged younger than 60 years with severe disability at hospital discharge remained mostly identical over time. Numbers indicate % of patients in the respective mRS category. (**B**) The cumulative number of mRS points at discharge (as a measure of the burden of stroke disability to bear for the caregiving system) remained identical over the analyzed decade from 2010 to 2019. Numbers indicate % of cumulative mRS points related to the respective mRS category.

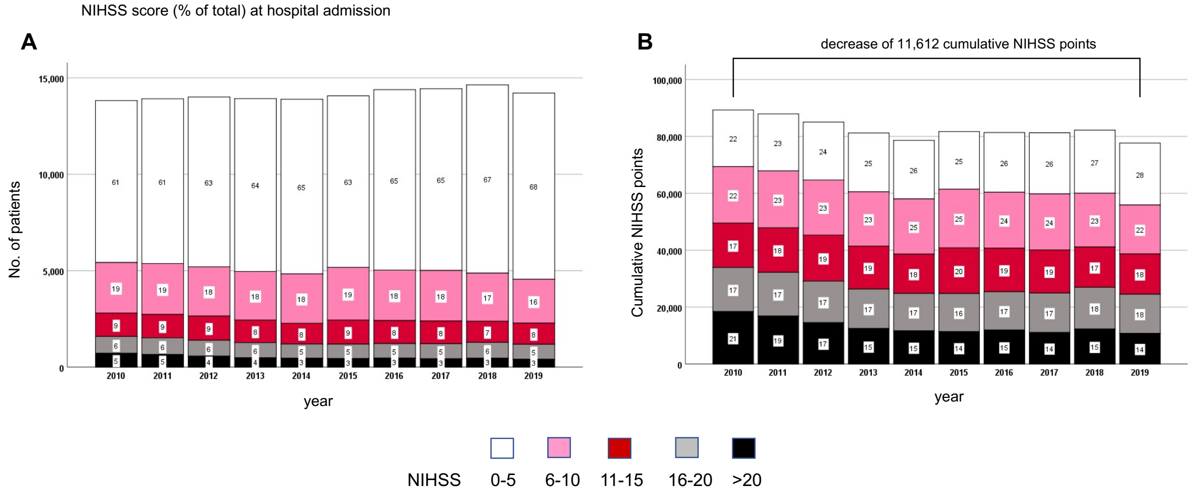

Supplementary Figure S7. (**A**) Whereas the total number of patients remained stable during the study period, the proportion of patients with severe clinical deficits at hospital admission decreased over time. Numbers indicate % of patients in the respective NIHSS category. (**B**) The cumulative number of NIHSS score points at hospital admission discharge (as a measure of the burden of stroke severity to bear for the treating hospitals) decreased over time. This was due to a reduction of severely affected cases. 11612 cumulative NIHSS score points were saved in the federal state of Hesse in 2019 in comparison to 2010. Numbers indicate % of cumulative NIHSS points related to the respective NIHSS category.

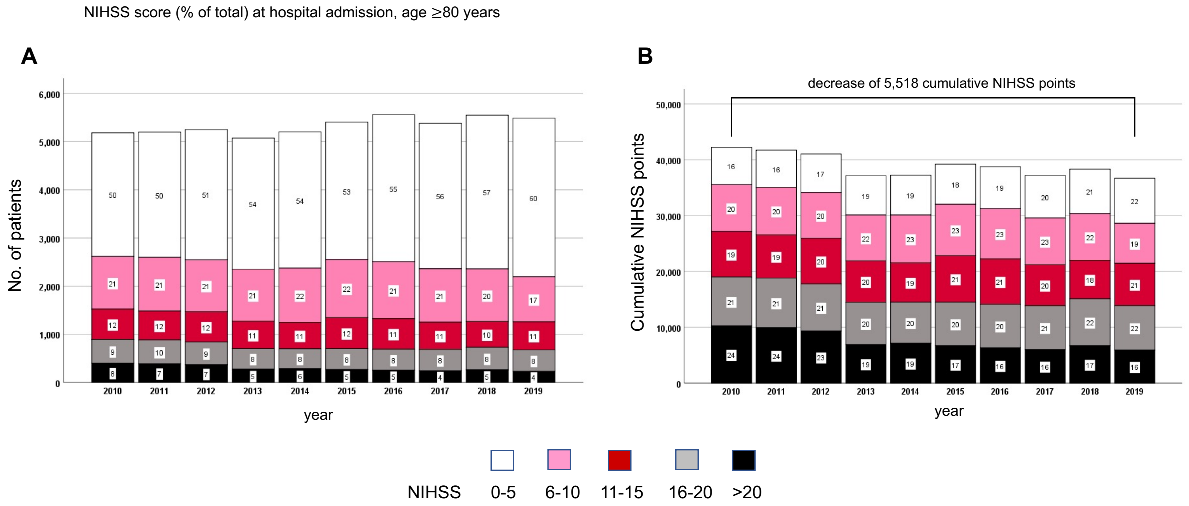

Supplementary Figure S8. The figure only shows data on patients aged 80 years or older at hospital admission. (**A**) The proportion of patients aged 80 years or older with severe clinical deficits at hospital admission decreased over time. Numbers indicate % of patients in the respective NIHSS category. (**B**) The cumulative number of NIHSS score points at hospital admission discharge (as a measure of the burden of stroke severity to bear for the treating hospitals) decreased over time. This was due to a reduction of severely affected cases. In total 5518 cumulative NIHSS score points were saved in the federal state of Hesse in 2019 in comparison to 2010 in patients aged 80 years or older alone. Numbers indicate % of cumulative NIHSS points related to the respective NIHSS category.

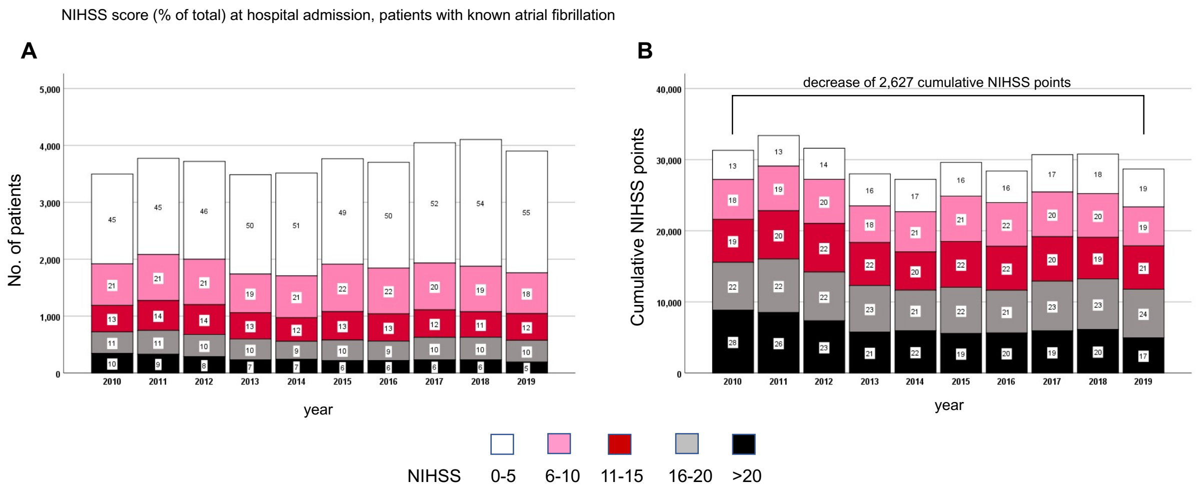

Supplementary Figure S9. The figure only shows data on patients with known atrial fibrillation at hospital admission. (**A**) The proportion of patients with known atrial fibrillation at hospital admission with severe clinical deficits at hospital admission decreased over time. Numbers indicate % of patients in the respective NIHSS category. (**B**) The cumulative number of NIHSS score points at hospital admission discharge (as a measure of the burden of stroke severity to bear for the treating hospitals) decreased over time. This was possibly due to a reduction of severely affected cases. In total 2627 cumulative NIHSS score points were saved in the federal state of Hesse in 2019 in comparison to 2010 in patients with known atrial fibrillation at hospital admission. Numbers indicate % of cumulative NIHSS points related to the respective NIHSS category.

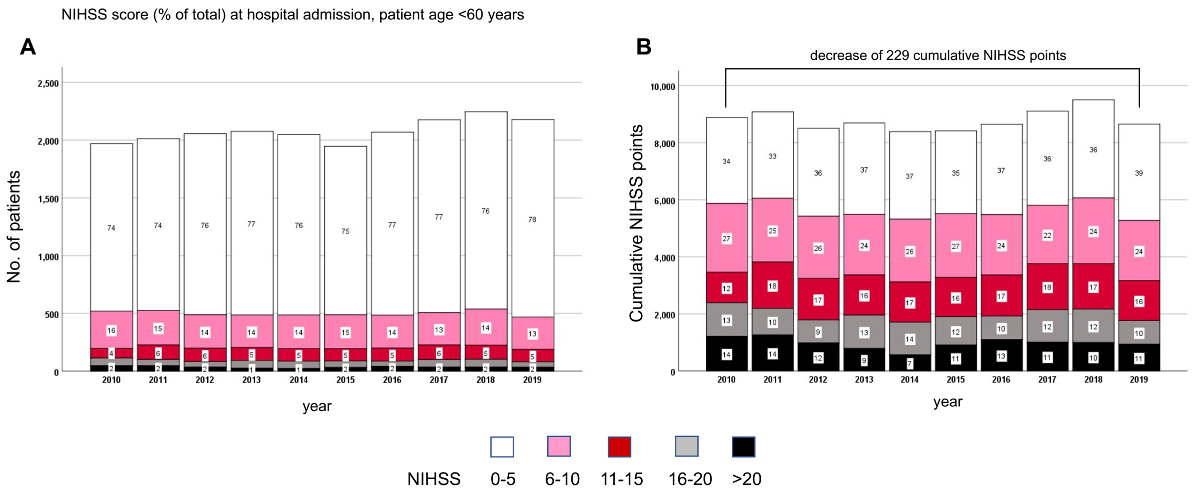

Supplementary Figure S10. The figure only shows data on patients aged younger than 60 years at hospital admission. (**A**) The proportion of patients aged younger than 60 years at hospital admission with severe clinical deficits at hospital admission remained mostly identical over time. Numbers indicate % of patients in the respective NIHSS category. (**B**) The cumulative number of NIHSS score points at hospital admission discharge (as a measure of the burden of stroke severity to bear for the treating hospitals) did not relevantly decrease over time. In total only 229 cumulative NIHSS score points were saved in the federal state of Hesse in 2019 in comparison to 2010 in patients with known atrial fibrillation at hospital admission. Numbers indicate % of cumulative NIHSS points related to the respective NIHSS category.
